# Nuclear Protein in Testis (NUT) Carcinoma With Nasopharyngeal and Intracranial Involvement: A Case Report

**DOI:** 10.7759/cureus.93889

**Published:** 2025-10-05

**Authors:** Natasha Holden, James Park, Chul Chae, Dassaev Vigil, Donald Dennis

**Affiliations:** 1 College of Osteopathic Medicine of the Pacific, Western University of Health Sciences, Pomona, USA; 2 Medical Imaging, Arrowhead Regional Medical Center, Colton, USA; 3 Radiology, Arrowhead Regional Medical Center, Colton, USA; 4 Internal Medicine, University of South Alabama, Mobile, USA

**Keywords:** brd4-nut, nasopharyngeal cancer (npc), nut carcinoma, nutm1-brd4 mutation, nut midline carcinoma (nmc)

## Abstract

Nuclear protein in testis (NUT) carcinoma is a rare and aggressive malignancy characterized by NUT gene rearrangement. Previously classified as NUT midline carcinoma, this malignancy was reclassified to NUT carcinoma in order to reflect that it can occur outside midline structures of the body. This case report follows a 43-year-old male presenting with a nasopharyngeal mass invading the frontal brain region, ultimately diagnosed as NUT carcinoma. Here, we detail the clinical and molecular characteristics of this tumor to contribute to the growing body of literature on this rare malignancy, while acknowledging potential diagnostic challenges and therapeutic considerations.

## Introduction

Nuclear protein in testis (NUT) carcinoma is an exceedingly rare, aggressive subtype of squamous carcinoma with a median survival of 6.5 to 9.7 months [1,2]. Characterized by rearrangements of the NUTM1 gene, NUT carcinoma most commonly arises when the NUTM1 gene fuses to the BRD4 transcriptional activator [3]. Several studies suggest the fused BRD4-NUTM1 oncogene acts as a single driver of growth and blockade of differentiation [3-5]. Alternative oncogene arrangements may occur [6-8], but the extent to which these arrangements contribute to NUT carcinoma pathogenesis remains uncertain and is less well understood compared to the BRD4-NUTM1 fusion. Previously known as NUT midline carcinoma due to its proclivity for midline structures such as the head, neck, or mediastinum, but renamed because it can occur at multiple sites, NUT carcinoma progresses rapidly [2]. While molecular testing and immunohistochemistry for NUT protein expression have advanced diagnostic accuracy, standard treatment regimens such as surgery, radiation, and chemotherapy offer limited effectiveness [1]. Promising research into bromodomain and extra-terminal (BET) inhibitors targeting the BRD4-NUT fusion protein is ongoing but remains experimental [9].

Moreover, the classification of these tumors is controversial and calls for the need to expand the current literature and gain a better understanding of how different clinical classifications may impart different treatment outcomes [10,11]. Despite contemporary research suggesting low tumor mutational burden and overall reliance on a single oncogenic driver [3,4], the true incidence of NUT carcinoma is likely underestimated due to its rarity and consequent increased potential for misdiagnosis/misclassification [5]. To this end, this case study aims to add to the increasing literature in an attempt to better classify and understand this treatment-resistant orphan disease. This report documents a unique case of NUT carcinoma with nasopharyngeal origin and frontal brain invasion, emphasizing the diagnostic challenges and therapeutic considerations.

## Case presentation

A 43-year-old male presented to the Arrowhead Regional Medical Center (ARMC) emergency department (ED) on March 3, 2024, complaining of worsening sinus pressure, sinus pain, and right V2 facial numbness. Of note, symptoms began approximately one month prior, when the patient had six tooth extractions due to fractured teeth, which were complicated by infection and treated with a course of amoxicillin and azithromycin. Two weeks after his dental procedure, the patient presented to urgent care with symptoms of facial pain, numbness, and swelling; the patient was prescribed clindamycin.

During his ED visit, the patient was well-appearing on exam, nontender, and nonseptic. Moreover, the patient’s vital signs were within normal limits, and labs were without significant derangement or leukocytosis. A maxillofacial CT scan with contrast was obtained and was unremarkable.

**Figure 1:**
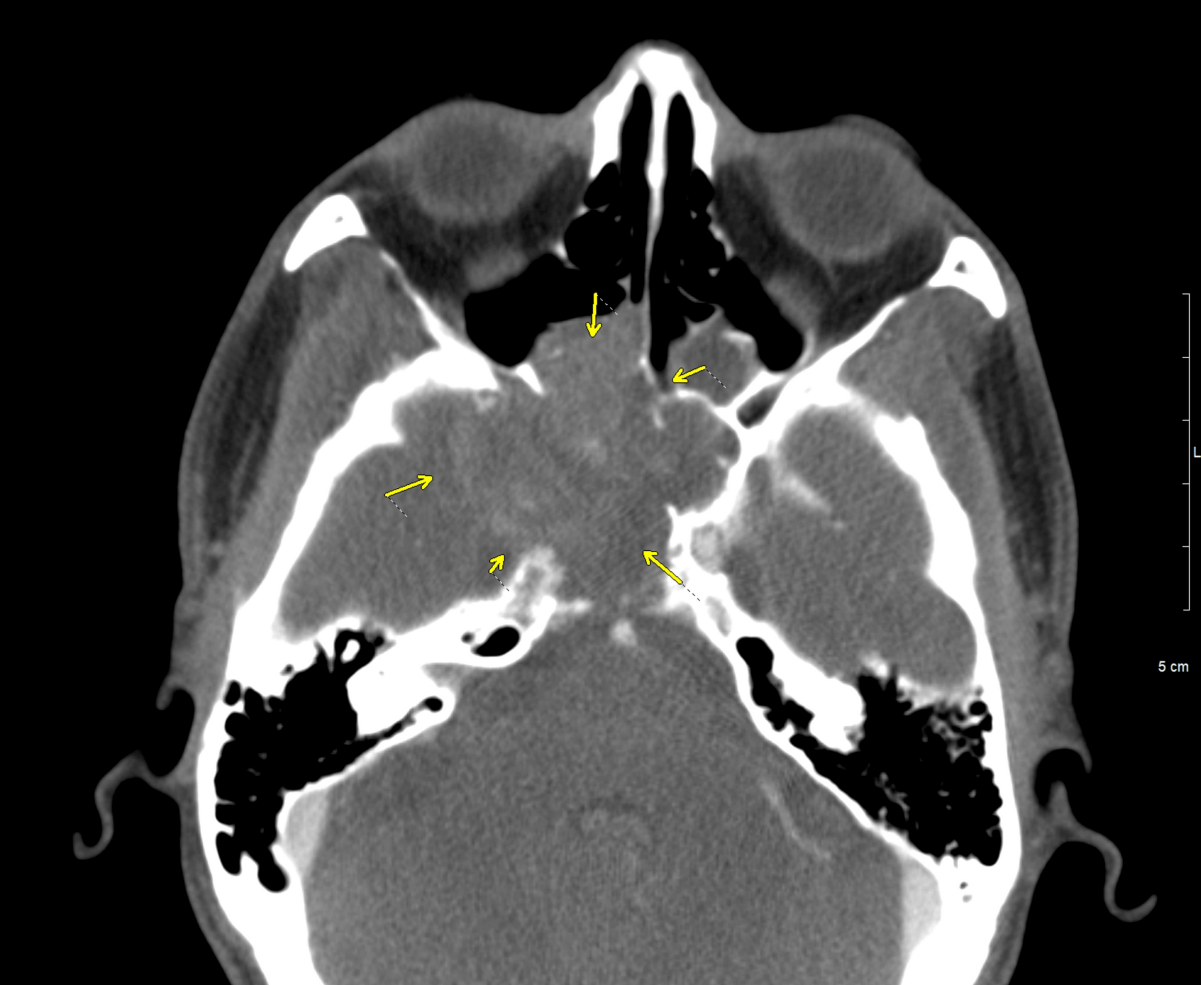
CT axial head showing large heterogenous mass (yellow arrows) centered within the ethmoid sinus with extension into right middle cranial fossa.

**Figure 2:**
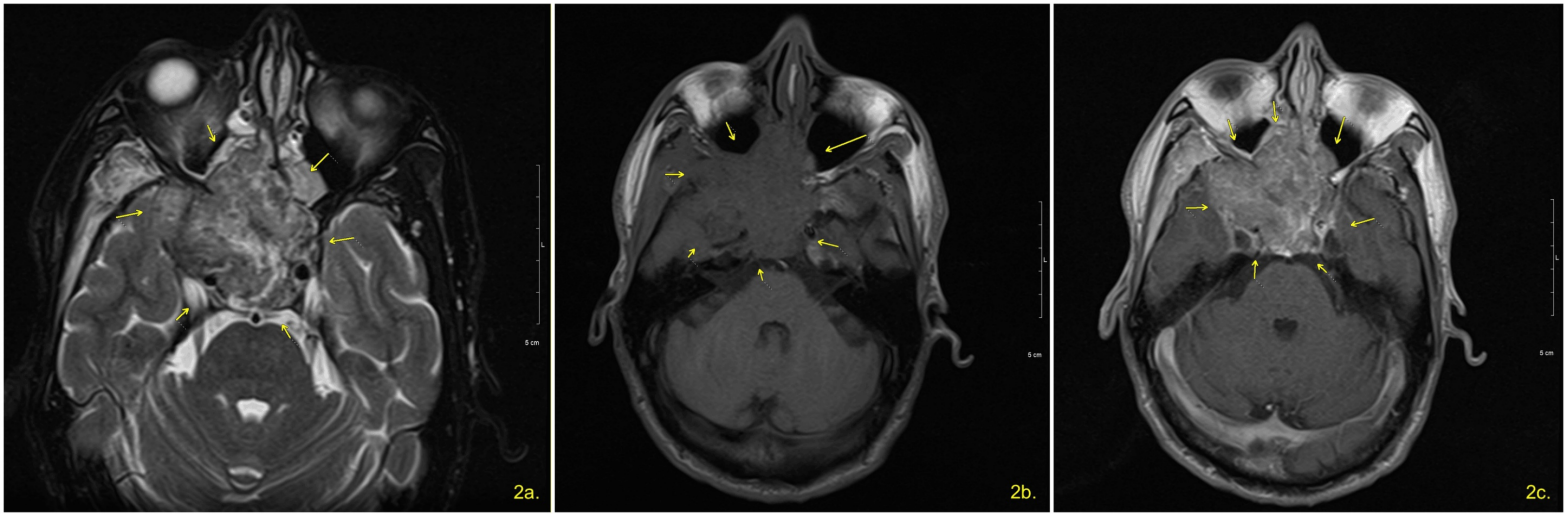
MRI appearance of a heterogeneous, enhancing mass centered within the ethmoid sinus, measuring approximately 6 x 4 cm, involving the mid-posterior ethmoid and sphenoid sinuses with gross destruction of the right greater wing of sphenoid and extension to the right temporal fossa. There is effacement of the right pterygopalatine fat and right Meckel's cave. (2a) Axial T2-weighted MRI demonstrates the mass as heterogeneously hyperintense (yellow arrows); (2b) axial T1-weighted MRI pre-contrast shows the mass as intermediate intensity (yellow arrows); (2c) axial T1-weighted MRI post-contrast demonstrates heterogeneous enhancement of the mass (yellow arrows)

An axial CT head, as seen in Figure 1, and an MRI orbit brain with and without contrast, as seen in Figure 2, were ordered and completed on March 26, 2024.

The patient underwent a bicoronal craniotomy for resection of the tumor on April 12, 2024. A tumor specimen was collected, and immunohistochemistry and chromogenic in situ hybridization were performed at Massachusetts General Hospital. Immunohistochemistry was positive for NUT (diffuse, nuclear), keratin MNF116, p16, INI1 (SMARCB1, retained), and Brg1 (SMARA4, retained).

Immunohistochemistry was negative for p63 (scattered cells), INSM1, chromogranin, synaptophysin, NKX2.2, and desmin. Chromogenic in situ hybridization was negative for Epstein-Barr encoded mRNA (EBER). Positive NUT (diffuse, nuclear) rules out other carcinomas lacking the NUT gene fusion and is a diagnostic hallmark of NUT carcinoma, confirming the diagnosis. 

The patient was referred to the neuro-oncology service at City of Hope, and a multidisciplinary treatment plan including radiation oncology and neurosurgery follow-up was initiated. However, on May 14, 2024, the patient and the patient’s family presented to neurosurgery for follow-up, indicating worsening of symptoms. Notably, the patient had stopped eating, desired to bathe continuously, and was engaged in other abnormal behavior. The family and patient decided to proceed with hospice care, and the patient passed away.

## Discussion

This case highlights the critical need for early recognition of NUT carcinoma in order to attempt urgent, personalized treatment strategies. While no formal guidelines specify testing protocol, several studies support performing NUT testing at the initial diagnostic evaluation for suspected cases [12-14]. Nasopharyngeal involvement, as seen in this case, is particularly rare; a multicenter case series of 30 head and neck NUT carcinomas identified only 3% of cases that involved the nasopharynx [14]. A separate study of undifferentiated carcinomas of the upper aerodigestive tract found that approximately 20% of Epstein-Barr Virus (EBV)-negative cases harbored NUT rearrangements, with several cases arising from the nasopharynx [15]. Therefore, it is important to consider NUT carcinoma in the differential diagnosis of undifferentiated nasopharyngeal carcinomas, particularly when EBV status is negative or morphology is atypical.

Luo et al. (2025) acknowledge that NUT carcinoma is a squamous cell carcinoma based on histopathological, transcriptional, and molecular cell of origin characteristics, arguing that the “orphan classification of NUT carcinoma as a distinct entity leads to a lack of awareness of this malignancy among oncologists and surgeons" [10]. Consequently, some research suggests NUT carcinoma has a higher estimated annual incidence than previously thought (exceeding the occurrence of some rare subtypes of head and neck squamous cell carcinoma) [10].

Given the underwhelming outcomes associated with current treatment regimens for NUT carcinoma, novel targeted therapies are currently being researched. BET inhibitors have shown promise in clinical trials [9,16,17]; BET inhibitors act by competitively inhibiting BRD4 function, thereby suppressing tumor growth [18]. Pathologically, the BRD4-NUT fusion protein recruits histone acetyltransferases like p300 to form hyperacetylated nuclear domains, which promote continuous expression of oncogenic transcription factors that block differentiation and promote uncontrolled proliferation [19]. Research suggests that, in conjunction with surgery [20] and potentially radiotherapy [21], these treatments may improve survival and merit further investigation.

## Conclusions

In summary, this case underscores the importance of considering NUT carcinoma in the differential diagnosis of aggressive nasopharyngeal masses. The severity and rapid progression of this carcinoma highlight the need for increased awareness for improving early diagnosis, and also necessitate consideration of reclassifying NUT carcinoma. As seen by this patient’s rapid disease progression, a multidisciplinary approach and advanced molecular diagnostics are essential for accurate diagnosis and management.
